# Tensile Notch Sensitivity of Additively Manufactured IN 625 Superalloy

**DOI:** 10.3390/ma13214859

**Published:** 2020-10-29

**Authors:** Gheorghe Matache, Alexandru Paraschiv, Mihaela Raluca Condruz

**Affiliations:** National Research and Development Institute for Gas Turbines COMOTI, 220D Iuliu Maniu Av., 061126 Bucharest, Romania; alexandru.paraschiv@comoti.ro (A.P.); raluca.condruz@comoti.ro (M.R.C.)

**Keywords:** IN 625, tensile notch sensitivity, notch strength ratio, additive manufacturing

## Abstract

The notch sensitivity of additively manufactured IN 625 superalloy produces by laser powder bed fusion (LPBF) has been investigated by tensile testing of cylindrical test pieces. Smooth and V-notched test pieces with four different radii were tested both in as-built state and after a stress relief heat treatment for 1 h at 900 °C. Regardless of the notch root radius, the investigated alloy exhibits notch strength ratios higher than unity in both as-built and in stress-relieved states, showing that the additive manufactured IN 625 alloy is not prone to brittleness induced by the presence of V-notches. Higher values of notch strength ratios were recorded for the as-built material as a result of the higher internal stress level induced by the manufacturing process. Due to the higher triaxiality of stresses induced by notches, for both as-built and stress-relieved states, the proof strength of the notched test pieces is even higher than the tensile strength of the smooth test pieces tested in the same conditions. SEM fractographic analysis revealed a mixed mode of ductile and brittle fracture morphology of the V-notched specimens regardless the notch root radius. A more dominant ductile mode of fracture was encountered for stress-relieved test pieces than in the case of the as-built state. However, future research is needed to better understand the influence of notches on additive manufactured IN 625 alloy behaviour under more complex stresses.

## 1. Introduction

Material failure is considered an important task during material selection and in the designing phase of a new product. Many studies have been conducted over the years regarding the characterization of material properties and failure modes. It is known that materials fail when they are subjected to loads beyond their limit, but sometimes material discontinuities are responsible for material failure. Material discontinuities such as cracks or notches are considered to be stress concentrators and they can be located externally (on a material’s surface) or internally (in a material’s mass). The assessment of notch influence on tensile properties is significant for material sensitivity determination [1].

Generally, the discontinuities can weaken the materials, but some studies reported that the notches can weaken brittle materials, but they can strengthen high ductile materials [2,3]. In the last few decades, different methods have been applied to study the influence of notches in several metallic, ceramic and composite materials and various analysis methods have been used such as share sliding theory, finite element analysis etc. [1,4,5,6,7,8].

As previously shown by several studies [9,10,11,12,13,14], there is no universally applicable theory for all materials, from ductile polycrystalline metals to fragile ceramic materials, with regard to the influence of notches on material strength. Many authors studied the influence of notch size and geometry on different alloys strength. Owolabi et al. [15] studied the influence of notch size and material microstructure on notch sensitivity for an IN 100 superalloy. They simulated a probabilistic mesomechanics model and compared it with experimental results and concluded that the probability of failure, fatigue notch factor and associated notch sensitivity index increases with the increasing notch root radius. Moreover, the grain orientation influences the IN 100′s fatigue strength. Sanyal et al. [16] studied the effect of notch geometry on fracture features of IN 625 and found out that different stress triaxialities induced by notch root radius variation lead to a change in morphological fracture features as well as in different mechanical properties.

Grain size effects on the notch sensitivity of pure aluminum was studied by Lorenzino et al. [17] Based on the results, it was concluded that the relationship between notch size and grain sizes govern the stress concentration and increases the grain size, while keeping the holes size fixed can reduce the influence of the notch on the fatigue strength. Increasing the size of the holes while keeping a fix grain size results in a decrease in fatigue strength, the finer the grain, the higher the strength decrease was noticed.

Peron et al. [18] investigated the tensile behaviour of notched specimens manufactured from a Ti-6Al-4V alloy using the Electron Beam Melting (EBM) technology. They showed that the presence of notches induces the increase of tensile strength of as-built material, and that the tensile strength increases with the severity of the notch, which was found to disagree with the results for a conventionally manufactured Ti-6Al-4V wrought alloy. They also showed that the notch strengthening behaviour of EBM material differs significantly from that previously reported for wrought alloys [19] and is related to the influence of process-induced defects on failure mechanism.

As additive manufactured materials are more and more researched [20,21], different studies were conducted to assess the notch sensitivity of such materials. Existing theories are less applicable for high anisotropic materials such as additively manufactured alloys, which exhibit different mechanical properties depending on the spatial building orientation [22,23,24]. Moreover, the additive manufactured metallic alloys inherently exhibit discontinuities such as porosities and micro cracks, as a result of manufacturing methods, acting as stress concentrators [25,26,27,28,29,30,31,32].

The aim of the present work was to assess by experiments the room temperature tensile notch sensitivity of an IN 625 superalloy produced by additive manufacturing from metal powder and to investigate the V-notch root radius influence on the stress triaxiality and the surface fracture morphology.

## 2. Materials and Methods

For the purpose of this study, IN 625 superalloy coupons were manufactured by selective laser melting using as feedstock IN 625 metal powder produced by vacuum induction melting inert gas atomization (particle size range 15–45 μm and cumulative distribution by mass recommended by the machine manufacturer *Q_3_*(*d*) [%]: *d*_10_ = 20 ± 2 μm, *d*_50_ = 30 ± 5 μm, *d*_90_ = 45 ± 5 μm) supplied by LPW Technology Ltd. (Runcorn, UK, subsidiary of Carpenter Technology Corporation). The chemical composition of the metal powder certified by the supplier is presented in Table 1.

**Figure 1:**
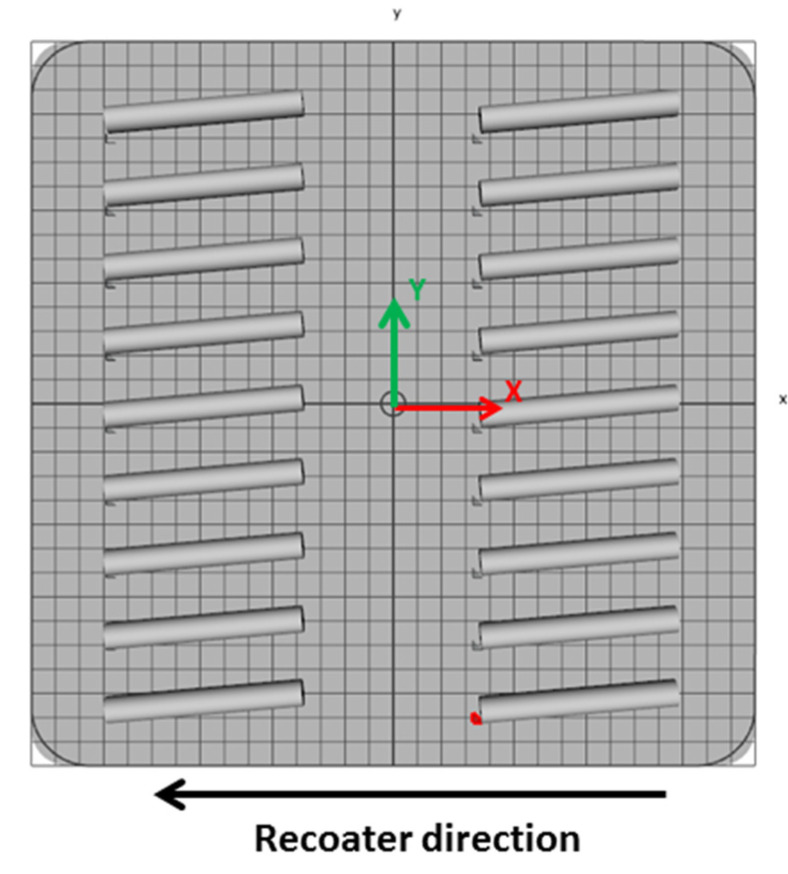
Positioning of the rods on the building plate.

**Figure 2:**
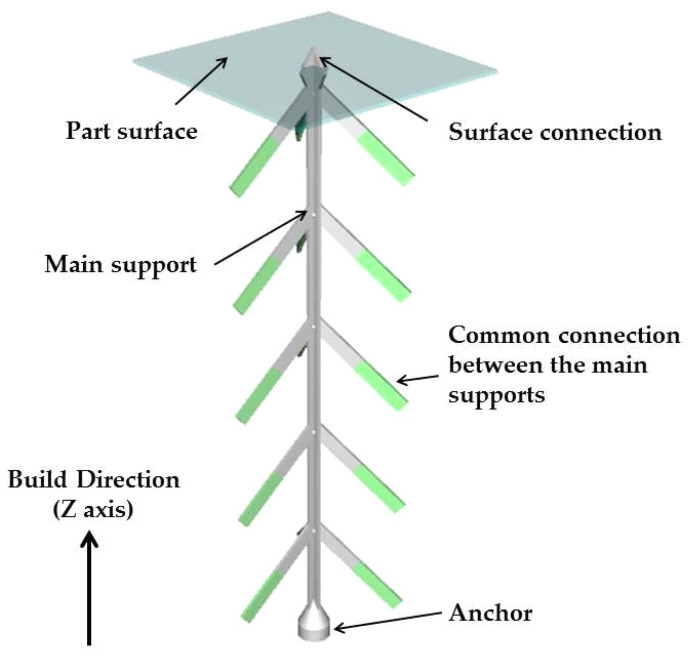
Geometry of the support structures.

Cylindrical rods of 11 mm in diameter and 80 mm long were manufactured using a Lasertec 30SLM machine (DMG MORI, Bielefeld, Germany). The machine is equipped with an ytterbium fiber laser and uses the laser powder bed fusion technique. All rods were manufactured in a horizontal position, along an *X*-axis, rotated with 5°, and using the same process parameters for hatching (laser current 2000 A, exposure time 40 µs, points distance 40 µm, layer thickness 50 µm, 90° scanning strategy rotated with 90° between two successive layers, and hatch distance 0.11 mm). The positioning of the rods on the building plate is presented in Figure 1. A cross-type support structure (Figure 2) with a height of 3 mm was designed using the machine software to support overhanging down-facing surfaces of the cylindrical rods. The support structure geometry consisted of four functional areas: main support (size 0.3 mm, boundary distance 2 mm), surface connection (type cross, size 0.8 mm, height 0.6 mm, surface offset 0.3 mm, surface angle 45°), connections between the main supports (type line, size 0.3 mm) and the anchor (type circle, size 0.8 mm and height 0.5 mm). The support structures were manufactured using the following process parameters: laser current 700 mA, exposure time 60 µs, points distance 30 µm, layer thickness 50 µm. The rods were manufactured on a building plate preheated and maintained throughout the process at a temperature of 60 °C.

**Figure 3:**
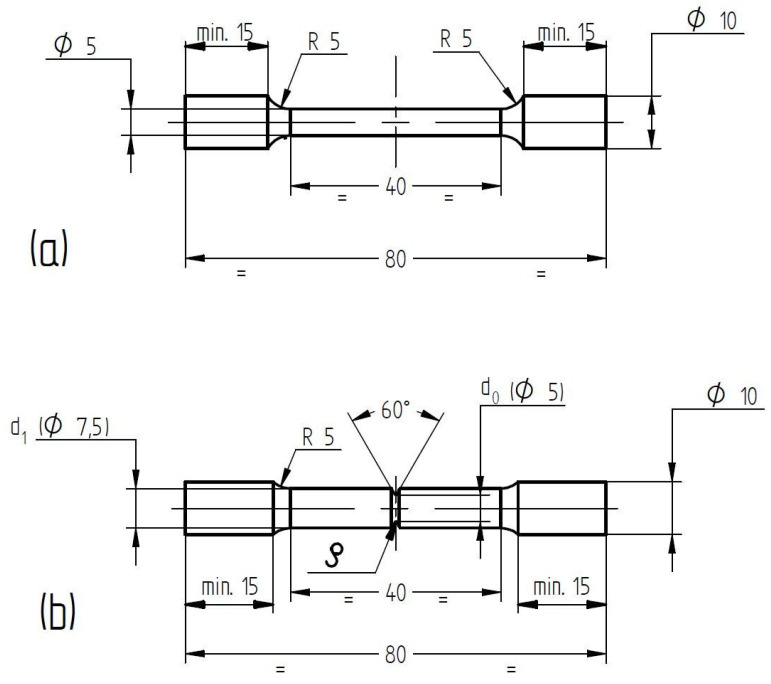
Tensile test pieces dimensions: smooth (**a**) and notched test pieces (**b**) (units in millimeters).

The notch sensitivity of the material was investigated using tensile test pieces machined from the additive manufactured rods, in as-built and in stress-relieved conditions. The stress relief heat treatment was done using a Nabertherm LH 30/14 air furnace (Nabertherm GmbH, Lilienthal/Bremen, Germany) and consisted in heating with 10 °C/min up to 900 °C, held for 1 h, followed by air cooling. The rods were heat treated on the building plate before detachment and machining. No further heat treatment was performed after machining the test pieces to avoid any oxidation of the notches. Sets of two smooth and circumferential V-notched tensile test pieces with four different notch root radii were produced and subjected to tensile testing. The dimensions of the tensile test pieces are presented in Figure 3.

The size of the smooth test pieces follow the requirements of EN ISO 6892-1:2009 for standard proportional test pieces with *Lo* = 5.65√*So*, where *Lo* is the gauge length and *So* is the initial cross section of the test pieces.

**Figure 4:**
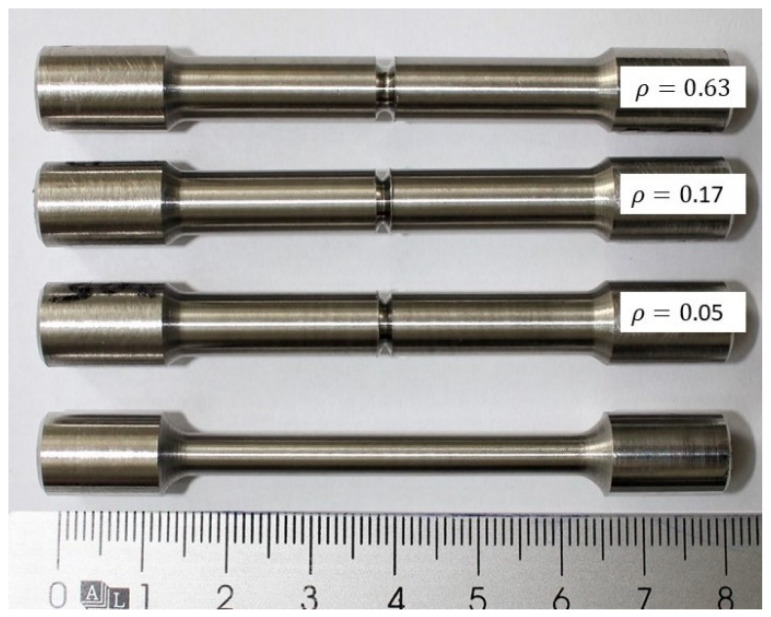
Machined smooth and notched tensile test pieces with different notch root radii.

The V-notches were machined with a CNC lathe EEN 320 CNC (Brunner Femmegmunkalo Ltd., Vecsés, Hungary) using carbide triangular turning inserts with a corner radii of 0.05 mm, 0.17 mm, 0.32 mm and 0.63 mm (TaeguTec Ltd., Daegu, Korea). The V-notches were machined at a speed of 200 rpm and a depth cut of 0.05 mm/rev. It was intended that the diameter at the notch root should be close to the diameter of the smooth test pieces (Ø 5 mm), so that the diameter of the cylindrical part of the notched test pieces was increased to 7.5 mm (Figure 3b). Figure 4 presents a photograph of machined smooth and notched tensile test pieces with different notch root radii.

Other authors have used the same approach to use notched specimens with the same notch root diameter of the minimum cross section as the smooth specimens [33].

Monotonic tensile tests were performed at room temperature according ISO 6892-1:2009, using an electromechanical universal testing machine, Instron 3369 Dual Column Testing System (Instron, Norwood, MA, USA), with a 50 kN load cell. Terms and definitions according ISO 6892-1:2009 are further used in this paper. During the tensile test, the strain rate over the parallel length was set to ${\dot{e}}_{Lc}$ = 0.00025 s^−1^ until the detection of the proof strength of 0.2%. After proof strength recording, the extensometer was removed and the strain rate over the parallel length was changed to ${\dot{e}}_{Lc}\;$= 0.0067 s^−1^.

Notch tensile testing was previously used to investigate high strength materials and embrittlement behaviour, as well as the notch sensitivity of high-temperature alloys through a Notch Strength Ratio (NSR) [33]. NSR values less than unity indicate that the material is notch brittle or notch sensitive, while supraunitary values characterize materials with different levels of plasticity.

Based on the experimental results, the NSR [34] and the stress concentration factor *K_t_* [35] were calculated according to Equations (1) and (2):

(1) $$K_{t}=1+2\sqrt{\frac{a}{\rho }}$$

(2) $$NSR=\;\frac{R_{m\left(N\right)}}{R_{m}}$$

where: *a* is the notch depth, *ρ* is the notch root radius, *R_m_*_(*N*)_ is the tensile strength of the notched test pieces and *R_m_* is the tensile strength of smooth test pieces.

The microstructure investigation was made using a light optical microscope Axio Vert.A1 MAT (Carl Zeiss Microscopy GmbH, Jena, Germany). The specimens were metallographically prepared by grinding and polishing and were etched with Aqua Regia reagent for 20 s.

The fracture surface morphology of the broken test pieces was investigated by scanning electron microscopy using a FEI Inspect F50 SEM (FEI Company, Brno, Czech Republic).

## 3. Results and Discussion

### 3.1. Notch Sensitivity

Actual size of the test pieces and tensile test results obtained on all smooth and notched test pieces are presented in Table A1 and Table A2 in Appendix A. The strength of AM IN 625, tensile strength and 0.2% proof strength, on smooth specimens in both as-built and stress-relieved conditions exceed the minimum requirements of ASTM F3056-14 for additive manufacturing of Nickel alloy with powder bed fusion [36]. The results obtained are also even greater than the requirements for the conventional hot-rolled alloy [37]. Based on the tensile test results, Table 2 and Table 3 summarize the calculated stress concentration factor and the notch strength ratio for both as-built and stress-relieved test pieces. To increase the accuracy of the calculations, the machined notch root radius (ρ), the specimen cylindrical part diameter (d_1_) and the initial diameter at the notch root (d_0_) were measured using a profile projector.

Conventionally, NSR values are used to differentiate between strengthening/weakening in static loading. The notch effect on the strength of different materials expressed by NSR differentiate materials into three categories. For conventional brittle materials, like ceramics, that exhibit NSR < 1, the effect is notch weakening, while supraunitary values of NSR designates materials for which the effect is notch strengthening (ductile crystalline materials). Intermediate values of NSR ~ 1, like some metallic glasses, are considered to be notch insensitive [1,38].

As can be seen, the investigated alloy exhibits notch strength ratios higher than unity in both as-built and stress-relieved states. Higher values were recorded for the as-built material as a result of the higher internal stress levels induced by the manufacturing process. However, the notch strength ratios for the IN 625 superalloy are very high in both cases, showing the notch strengthening effect on the material in the presence of stress concentrators, as circumferential V-notches.

**Figure 5:**
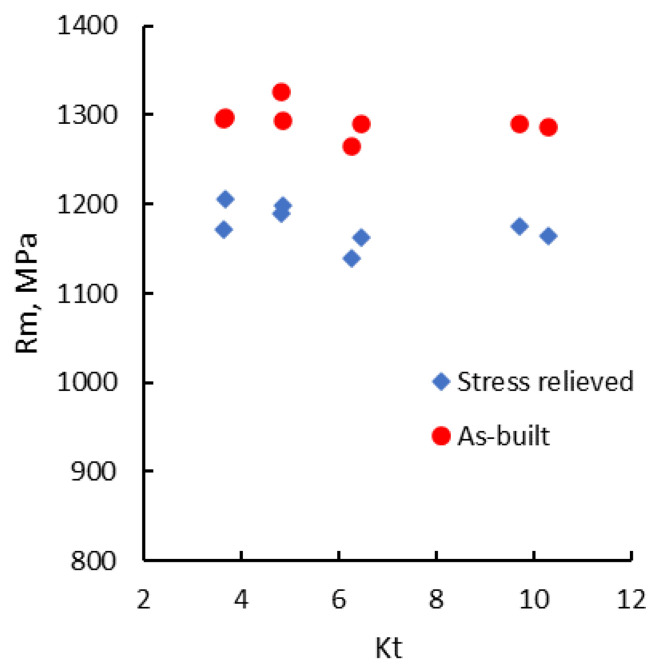
Tensile strength vs. the coefficient of stress concentration.

**Figure 6:**
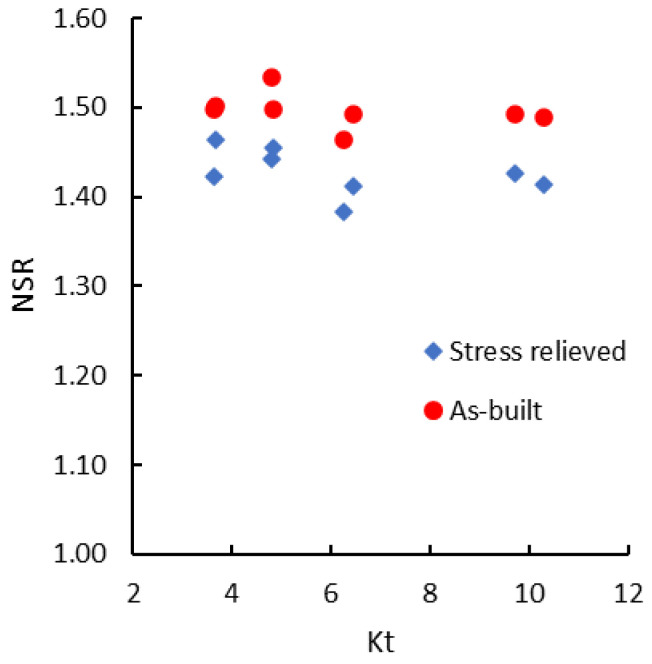
Notch strength ratio vs. the coefficient of stress concentration.

The analysis of the experimental data shows that in the range of the notch radii used, the stress concentration factor (Kt) slightly influences both the tensile strength and the notch strength ratio. Both Rm and NSR tend to decrease very slightly with the increase of Kt. Due to the scattering of Rm results, a linear regression cannot be established with sufficient statistical confidence. As depicted in Figure 5, the tensile strength of notched specimens tends to decrease as the stress concentration factor increases for both as-built and stress-relieved states. The same trend is also encountered in the case of the notch strength ratio (Figure 6).

This behaviour is consistent with other author findings for ductile engineering materials. Qu et al. [1] have studied the effect of the stress concentration factor on the strength of a Zr-based metallic glass compared to other materials like ceramics (Al_2_O_3_) and engineering materials (AZ80A Mg alloy and C45 steel). They show that all materials follow a similar tendency of NSR to decrease with increasing Kt. They also show that for the ductile engineering material (C45 steel), NSR decreases much more slowly in the investigated Kt range (2.5–5.5), as compared with the other materials.

Solberg et al. [39] have recently investigated the effect of defects and notches in quasi-static and fatigue loading of an alloy from the same class (Inconel 718) produced by selective laser melting. Their results on specimens tested in as-built conditions also showed NSR values higher than unity, the higher strength of the notched specimens being attributed to the notch strengthening effect.

In another recent study, Sinha et al. [38] investigated and compared the notch-tensile behaviour of conventional IN625 with 304 stainless steel, Ti–6Al–4V and Al0.1CrFeCoNi HEA alloys using V-notch geometry. For the conventional manufactured IN 625 alloy in annealed condition they reported an NSR value of 1.34, close to the results obtained in the present study on AM IN 625 in stress-relieved conditions that led to values of NSR in the range 1.38–1.43. These values indicate that in both conventional and AM IN 625, the notches have a strengthening effect on material.

An analysis of variance (ANOVA) was performed to assess the influence of the two identified factors on the variance of the response. The input data for ANOVA analysis are presented in Appendix B, Table A3 and Table A6. First, a Two-factor without replication ANOVA was carried out, the two considered factors being the specimen’s condition (as-built or stress-relieved), and the stress concentrator factor, Kt. The response was the tensile strength, Rm. The results are presented in Table A4. The analysis shows that both factors are statistically significant, even though the significance of Kt is considerably less. The *p*-value for the specimen’s condition is of the order of 10^−7^, while for Kt it is about 0.025, when α is 0.05. The F-test yields similar results. For Kt, the statistical significance is reflected by F = 4.96 > F critical = 3.78, and for the specimen’s condition F = 448.74 > F critical = 5.59.

Considering the impact of each factor on the total variance, ANOVA shows that the specimen condition accounts for about 91.5% of the variance, and Kt accounts for an additional 7%. The remaining error is quite small, with only about 1.5% of the total variation in Rm unaccounted for. This shows a very good identification of the factors affecting the response variability. To further assess the impact of each factor on the variability of the response, as the previous analysis had shown that they are both statistically significant, One-factor ANOVA were carried out. Taking into consideration the factor with the most significant effect alone, the specimen’s condition, the one factor ANOVA (Table A5) shows that both the p and the F tests continue to exhibit a high statistical significance, with a *p*-value of the order of 10^−9^, when α is 0.05, and F = 150.4 > F critical = 4.6. The 91.48% of variability explained by the considered factor remains.

If Kt is considered as a unique factor, its statistical relevance over the entire population is overshadowed by the much stronger influence of the specimen condition, as shown in Table A7. However, since it was already proven that there is statistical evidence that the specimen condition creates two distinct population categories, the One-factor Kt-based ANOVA was applied separately for the as-built, and the stress-relieved specimens (Table A8 and Table A9). This way, the factor recovers its statistical significance, with extremely low *p*-values (order of 10^−26^ for the as-built specimens, respectively 10^−24^ for the stress-relieved condition). Similarly, the F-tests result in F values four orders of magnitude over the critical threshold.

Furthermore, in both cases, the result variability is explained by Kt in a percentage of over 99.9%. It is important to keep in mind that the values refer to two variabilities after the segregation of data into two populations is discriminated by the sample condition. Thus, it can be concluded that Kt is a relevant factor with respect to the tensile strength, but its significance is obscured by the much stronger effect of the specimen’s condition. Once this effect is removed, Kt’s statistical relevance becomes very clear.

Finally, the correlation factor between Kt and Rm was determined for each specimen condition-based population (Table A10).

The results of the analysis show a medium strength correlation (0.35–0.44) between the Kt factor and the Rm response if the population is split in advance, depending on the condition of the specimens.

**Figure 7:**
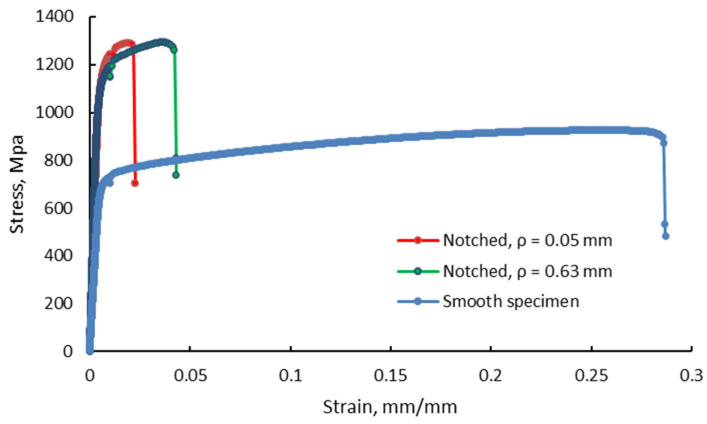
Recorded stress-strain curves for as-built smooth and V-notched test pieces (radii 0.05 mm and 0.63 mm).

The tensile stress-strain curves indicate that the tensile strength of smooth specimens is significantly lower when compared to the tensile strength recorded for V-notched test pieces. Contrariwise, the smooth test pieces exhibit a higher elongation at break. The tensile behavior of smooth and V-notched test pieces with the smaller and the larger notch root radius, in an as-built condition, is presented in Figure 7. There can be noticed a significant difference regarding the elongation at the break of smooth and V-notched specimens.

**Figure 8:**
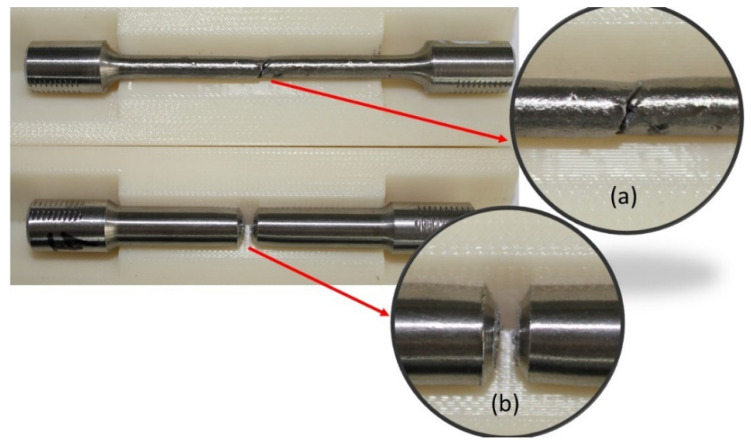
Comparison of the failure mode of stress-relieved smooth (**a**) and V-notched specimens with a notch root radius of 0.05 mm (**b**).

As expectation for ductile materials, during tensile loading, was that the smooth test pieces would elongate and that necking would occur before the fracture. Unlike this, the V-notched test pieces broke at the notch root, transverse to the applied load direction, without obvious deformation. Figure 8 shows comparative photos of broken smooth and V-notched IN 625 additive manufactured test pieces, highlighting the fracture behaviour under tensile load. The difference in elongation at the fracture of the two types of test pieces is very clear.

**Figure 9:**
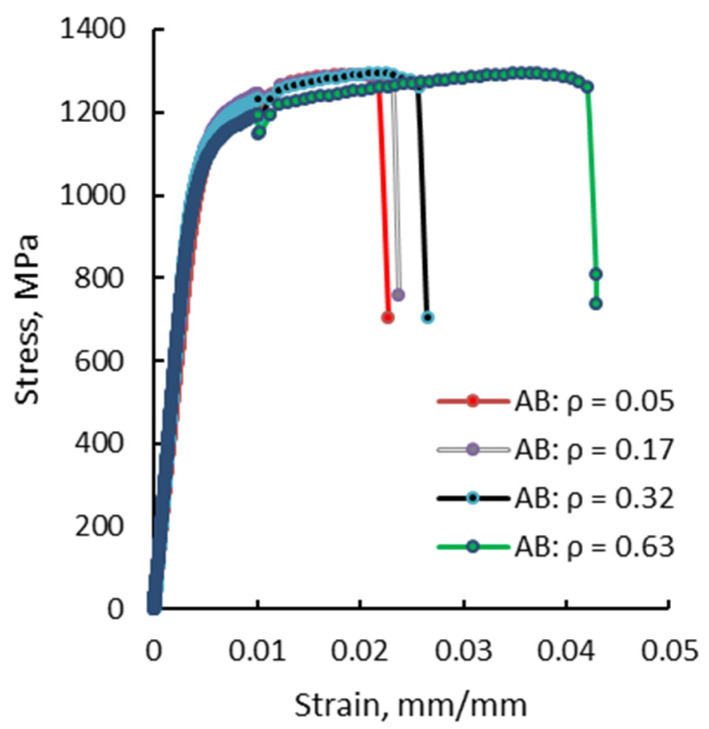
Stress-strain curves recorded for test pieces with different notch root radii tested in as-built condition.

In turn, the root notch radii caused different behaviors of the test pieces during tensile testing. Figure 9 presents the stress-strain curves recorded for notched specimens in as-built condition, with different notch root radii and indicates an increase in tensile strength along with a decrease in elongation at the break as the notch radius decreases. The increasing tensile strength is explained here by the appearance of a hardening effect in the cross section where the notch is located. The notch strengthening behavior was also encountered by other authors in the case of other materials such as Ti-6Al-4V [22].

**Figure 10:**
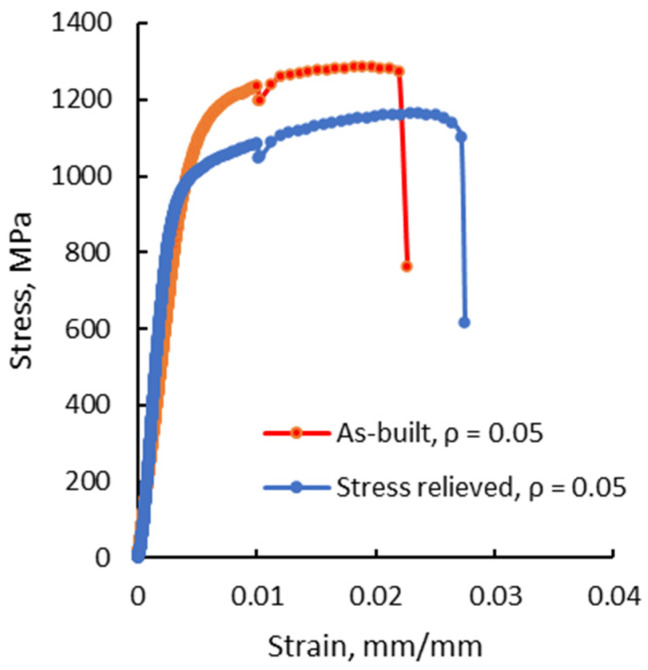
Stress-strain curves for V-notched test pieces with 0.05 mm notch root radius in as-built and stress-relieved states.

However, lower tensile strength values for the stress-relieved test pieces were recorded in comparison with the as-built state, due to the higher material plasticity. Figure 10 presents the stress-strain curves of two specimens with the sharpest notch root radius in both as-built and stress-relieved states. For the stress-relieved specimen, higher elongation at the break is recorded, but a lower tensile strength, as compared with the specimen in the as-built condition. Besides, plastic deformation is the only characteristic observed at the macroscopic level, revealed by the stress-strain curves. The higher plasticity of the stress-relieved specimens results in lower levels of tensile strength than in the as-built condition, associated with higher plastic deformations and elongations at the break in both smooth and notched specimens.

**Figure 11:**
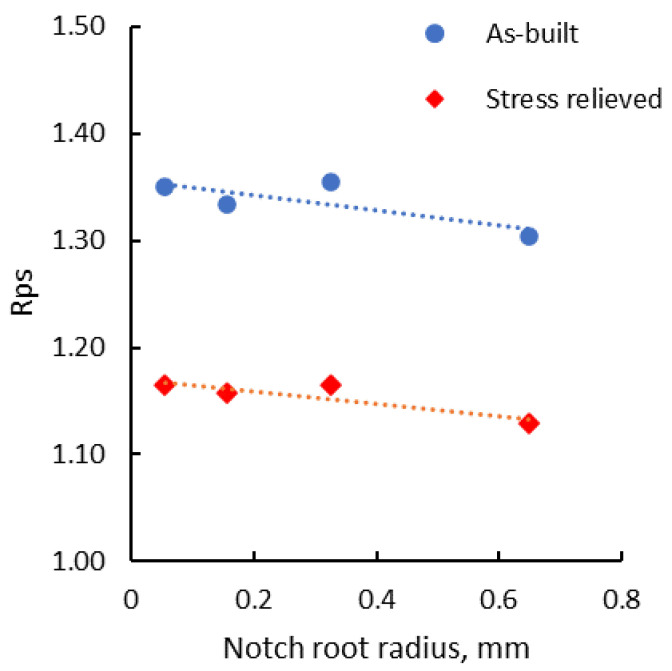
Normalized proof strength of notched test pieces as a function of notch root radius.

In all cases, the recorded proof strength of the notched test pieces was even higher than the tensile strength of the smooth test pieces tested in the same conditions. Similar behavior was also noticed in the case of stress-relieved test pieces. The proved strength of notched test pieces was normalized to the tensile strength of smooth specimens (Rps), which obviously shows this behavior (Figure 11). The normalized proof strength of notched test pieces in the as-built state is 30–35% higher than the tensile strength of the smooth test pieces, while in the stress-relieved state, this decreases to around 15–16%.

**Figure 12:**
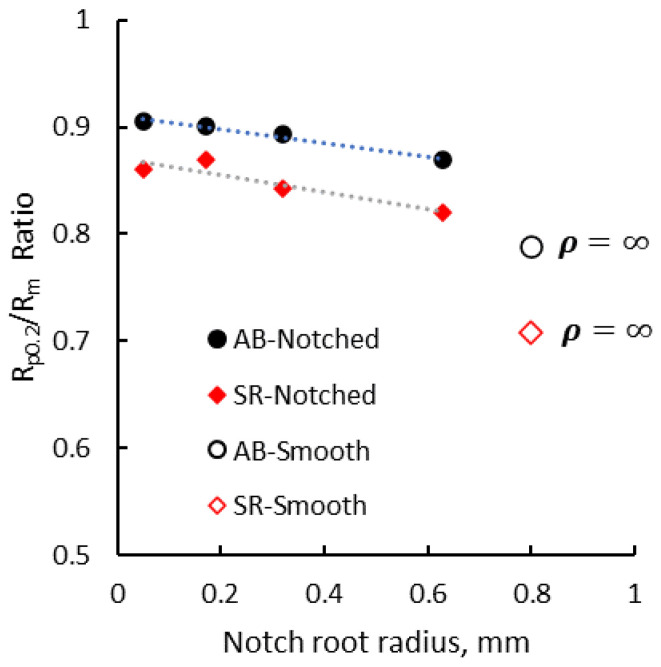
R_p02_/R_m_ ratio of V-notched and smooth specimens vs. notch root radius.

Experimental results have also shown that notch root radius influences the ratio between the proof strength and the tensile strength (R_p02_/Rm). Figure 12 presents the R_p02_/Rm ratio of V-notched and smooth test pieces as a function of the notch root radius, where *ρ* = ∞ points are the values recorded for the smooth specimens. The V-notch specimen’s tensile strength is a result of the ratio between the applied stress to the cross-sectional area at the notch root. As the root radius increases, the stress and strain are concentrated in a higher cross-sectional area resulting in a decrease of the mechanical strength for both as-built (AB) and stress-relieved (SR) test pieces. The R_p02_/Rm ratio of the smooth specimens is even lower.

The section of the true strain in the case of V-notch specimens is very narrow and is limited by the notch root, so the elongation at the break of the V-notch specimens is lower than that of the elongation of the smooth specimens, as can be observed in stress-strain curves. The value of the strain and the elongation at the break are dependent on the notch root radius as well, both elongation and strain increase as the notch radius increases.

It can be concluded that in both conditions, as-built and stress-relieved, tensile and proof strength values of the V-notched test pieces are higher than the values recorded for smooth test pieces and depend on the notch root radius. Due to a higher plasticity, the heat-treated test pieces exhibit lower strength as compared with the as-built specimens.

Likewise, the notch sensitivity ratio is lower in the heat-treated state than in the as-built state, but is still much higher than unity, showing that the additive manufactured IN 625 is not prone to brittleness induced by the presence of V-notches. Higher values of the tensile strength of notched specimens are generated by a higher triaxiality of stress, which leads accordingly to the increase of the proof strength and to a notch-strengthening behavior.

### 3.2. Stress Triaxiality

Tensile tests performed on round or flat test pieces with different notches are often used to study the influence of stress triaxiality on fracture strain or the combined influence of stress triaxiality and strain rate on material’s behaviour [40].

**Figure 13:**
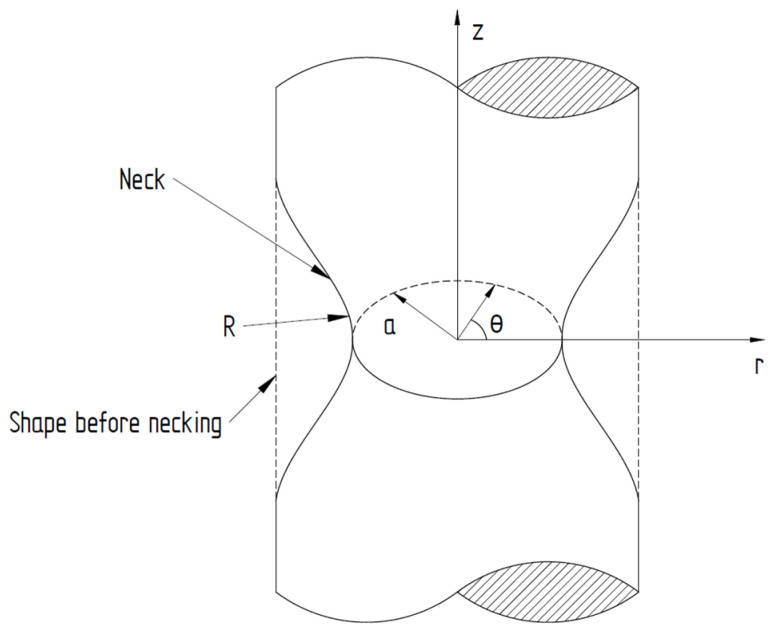
Tensile necking in a cylindrical test piece [43].

For notched round tensile test pieces, the stress triaxiality on the axis (Equation (3)) can be expressed as a function of the external surface curvature and the radius of the minimum cross-section [41,42].

(3) $$\eta =\;\frac{1}{3}+\ln\left(1+\;\frac{a}{2R}\right)$$

where *a* is the radius of the necking cross-section and *R* is the radius of the neck as depicted in Figure 13.

When dealing with cylindrical test pieces under tensile loads, it is very difficult or quite impossible to measure accurately *a* and *R*, as they continuously change during the test before the fracture initiation. For this reason, for the purpose of this paper, the initial stress triaxiality was calculated and used for notch sensitivity assessment of the additive-manufactured IN 625.

Using the dimensions of the test pieces before and after the tensile tests, summarized in Appendix A, both the initial stress triaxiality and the equivalent strain to fracture can be analytically calculated for the different notch root radii used. The initial stress triaxiality was calculated using Equation (3), where the radius of the neck (R) was considered to be the radius of the notch root, while the initial stress triaxiality of smooth cylindrical test pieces correspond analytically to *η* = 1/3.

The equivalent strain at fracture in the necking cross section of the round test pieces can be calculated using Equation (4), assuming that the strain is constant across the minimum cross-section [41].

(4) $${\bar{\varepsilon }}_{f\;}=2\cdot \ln\left(\frac{a_{0}}{a_{f}}\right)$$

where *a*_0_ is the initial radius of the round test piece, and *a_f_* is the final cross section radius of the test piece at fracture.

**Figure 14:**
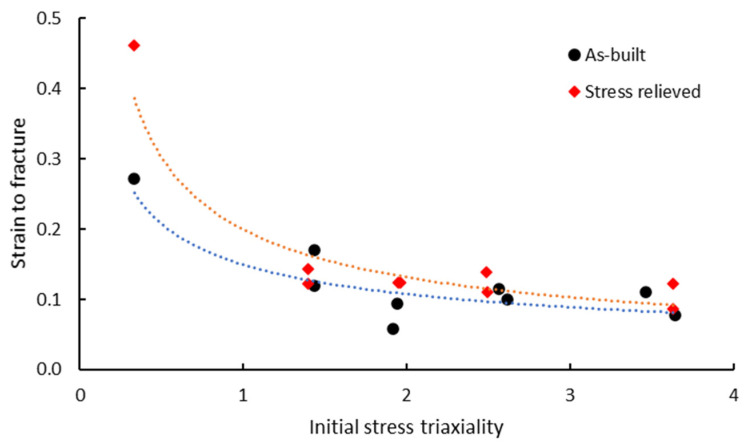
Equivalent strain to fracture as a function of initial stress triaxiality calculated based on experimental results.

The equivalent strain to fracture calculated from test results using Equation (4) as a function of initial stress triaxiality is presented in Figure 14.

As shown in Figure 14, various notch root radii generate different stress triaxialities. The smallest notch radius results in the highest stress triaxiality. As the stress triaxiality increases, the equivalent strain to fracture exponentially decreases. As a consequence, the ductility of material decreases with stress triaxiality, as was also previously shown by tensile stress-strain curves recorded for notched test pieces. Accordingly, the high stress triaxiality of notched test pieces results in higher tensile strength as compared with the smooth test pieces and generates supraunitary NSR values.

### 3.3. Microstructure

**Figure 15:**
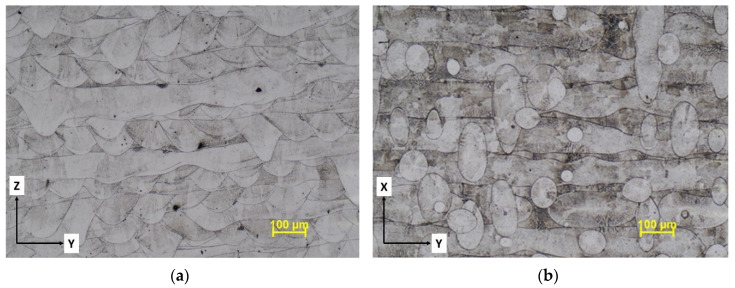
Microstructure of AM IN 625 in as-built condition parallel (**a**) and orthogonal to the build direction (**b**).

The microstructure of as-built IN 625 manufactured by LPBF exhibits characteristic features generated by the layer-by-layer building process. Figure 15 shows the optical micrographs of the as-built IN 625 taken in the YZ plane (parallel to the build direction) and in the XY plane (orthogonal to the build direction). In the YZ plane, the microstructure exhibits melt pools aligned perpendicular to the build direction overlapped over multiple tracks of the laser beam, typical for LPBF processing. Some inherent defects induced by the process, as porosities, are also visible. The YZ plane is transverse to the loading direction during tensile testing. In the micrograph taken in the XY plane, Figure 15b, the 90° hatching strategy is clearly visible.

**Figure 16:**
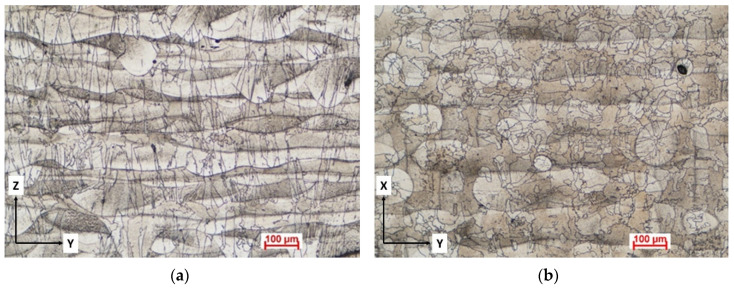
Stress-relieved microstructure showing the epitaxial columnar grain morphology in the YZ plane (**a**) and recrystallized grains in the XY plane (**b**).

The developed grain structure in the stress-relieved condition appears to be columnar with respect to the build direction and random in the plane orthogonal to the build direction. After the stress relief heat treatment for 1 h at 900 °C, the initial columnar grains grown along the *Z*-axis, transverse to the build direction, due to the thermal gradients induced by the additive manufacturing process, are still visible (Figure 16a). During the AM process, such epitaxial elongated grains grow aligned to the <001> direction across several layers. Orthogonal to the build direction, in the XY plane (Figure 16b), an incipient grain recrystallization is visible, but far from complete at the stress relief temperature.

Figure 15 and Figure 16 clearly show that in both as-built and after-heat treatment, the alloy exhibits pronounced microstructure anisotropy, which results in mechanical property anisotropy, caused by the loading direction relative to the building orientation. However, it is not the purpose of this paper to analyze the anisotropy in grain structure and its relationship with the mechanical properties of the material. A deeper analysis of the microstructural and tensile properties and anisotropy of Selective Laser Melting in manufactured IN 625 is presented elsewhere [44].

The high tensile properties of the AM IN 625 alloy in an as-built condition is a result of the very high solidification rates encountered in the LPBF process which, apart from generating very fine dendrites with a high dislocation density and a nanometric MC carbide, suppresses the macrosegregation of carbides or of the detrimental Laves phases and induces high thermal residual stresses. Post-processing by heat treatment aims to reduce residual stresses, to promote grain growth and phase precipitation, and to generate the appropriate mechanical properties to industrial requirements [45].

### 3.4. Fractographic Investigation

In order to have a good assessment of the notch sensitivity of Additively Manufactured IN 625, a fractographic investigation into the fracture surfaces of notched and smooth test pieces was performed. Both as-built and heat-treated test pieces were considered.

The existing literature presents various types of process-induced microstructural defects occurring in additive manufacturing metallic materials like gas porosity, lack-of-fusion (LOF) porosity, keyhole porosity and un-melted particles. Despite the fact that some of these defects can be avoided by optimizing the process parameters in order to obtain the highest possible densification, the existence of others is unavoidable due to the layer-by-layer manufacturing process and can lead to anisotropic mechanical properties. If the loading direction is parallel to the building direction, due to the inherent presence of the defects between successive layers, the load-bearing cross-section is reduced, and thus the tensile strength is lower than if the stress were applied transverse to the growth direction [44]. For specimens manufactured horizontally, as in the case of the present study, during tensile testing the load is applied along the built layers, orthogonal to the columnar grains’ growth direction with influence on material ductility. Ni et al. [46] studied the anisotropy of ductility in AM IN 718. They showed that ductility is controlled by the different cracking mechanisms induced by the tensile load direction relative to the grain boundaries. Loading perpendicular to the columnar grain-boundary (specimens built in a horizontal position) comply with a Mode I crack opening tension and leads to a lower ductility as compared with loads applied parallel to the columnar grain boundaries (specimens built along the *Z*-axis).

Nguejio et al. [47] have investigated the microstructure and the tensile properties of LPBF-manufactured IN625 in comparison with the conventional forged Inconel 625. By EBSD analysis they showed a very strong texture with a preferential orientation <001> of LPBF specimens in the YZ plane associated with the elongated grains. They also showed that the mechanical properties of the as-built specimens are higher than those obtained in heat-treated condition, consistent with the results obtained in this study. Similar results were reported for tensile strength and 0.2% proof strength on smooth specimens in both as-built and after-heat treatment specimens for 1 h at the same temperature of 900 °C. In respect of the effect of texture and loading direction, they showed that loading perpendicular to the elongated grains in the preferred <001> orientation, which corresponds to the YZ plan in this study, favors a faster activation of the micro-plasticity sites.

The results on notch effect of this study shown that the notches have influence not only on the tensile strength, but also on the tensile deformation. As shown in Figure 7, there is a significant difference regarding the elongation at break of the smooth and V-notched specimens. The material plasticity and the elongation at the break decreases as the stress concentration increases. Qu et al. [1] have previously shown that the plastic deformation is controlled by dislocation slipping homogenously over the whole gauge length of the smooth test pieces, and explains the higher tensile plasticity. As a result, during tensile loading, the smooth test pieces elongate much more than the notched specimens, and necking occurs and fracture is produced in the minimum cross-section. The stress concentration in notched test pieces restricts the plastic deformation at the notch root and leads to reduced ductility.

Brenne and Niendorf [48] have also shown that lower ductility in the presence of notches is caused by the concentration of deformation at the notch plane, while the outer areas barely deform.

Solberg and Berto [39] studied how the defects and geometrical notch features interact on fracture behaviour of materials and developed a diagram applicable to different loading cases. They have shown that for ductile engineering materials under static loading, the global dimensions are more dominating than the local defects. However, Lei et al. [2] showed that as a result of the geometrical constraints of the notch geometry, the strengthening effect is caused by forcing a transition from a shear-mode failure to a normal-mode failure.

**Figure 17:**
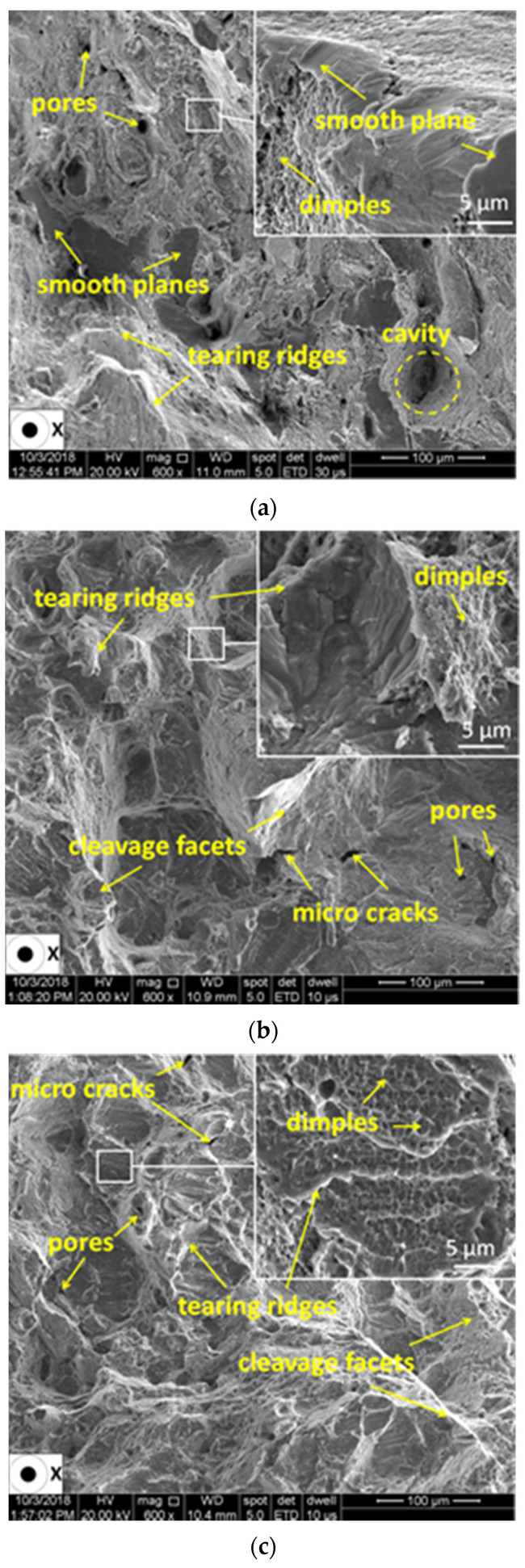
Fractographs of smooth (**a**) and V-notched test pieces. With root radii 0.05 mm (**b**) and 0.63 mm (**c**) tested in as-built state.

**Figure 18:**
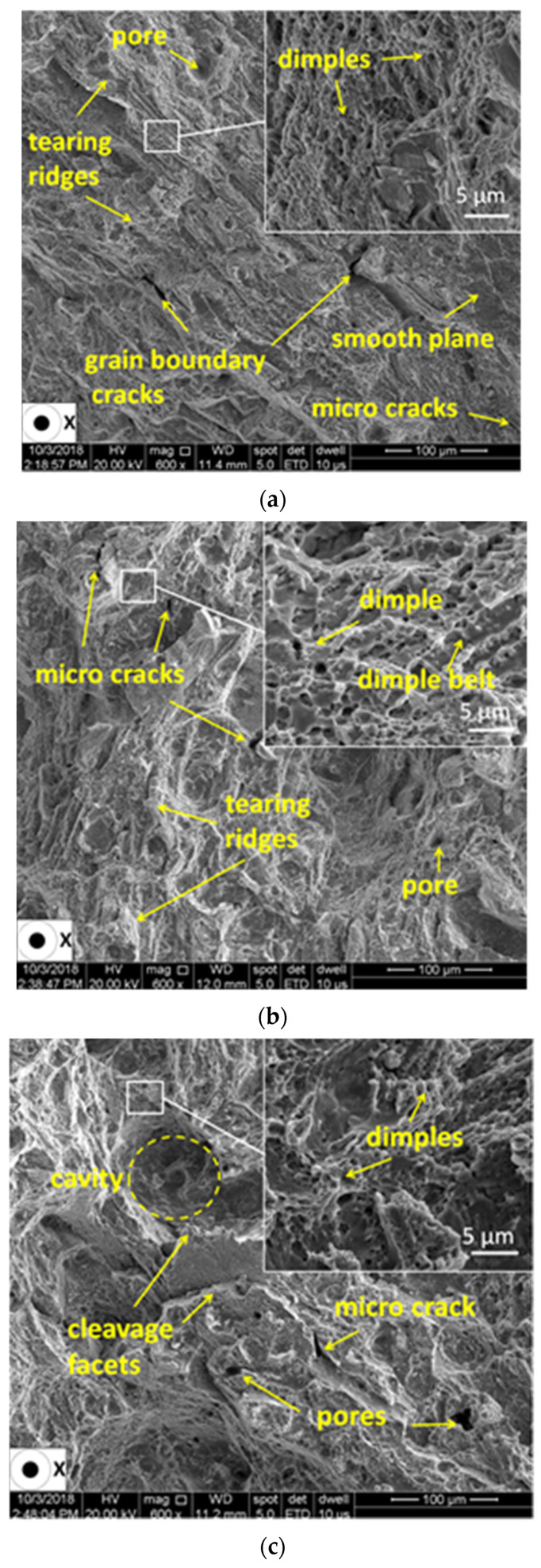
Fractographs of a smooth test piece (**a**) and a V-notched test piece. With root radii of 0.05 mm (**b**) and 0.63 mm (**c**), tested in the stress-relieved state.

The failure mechanism encountered for all test pieces is generally a mixed mode of ductile and brittle failure. SEM investigation revealed both the effects of notched and heat treatment on the fracture surfaces of test pieces. Figure 17 and Figure 18 present the SEM fractographs of the smooth and V-notched test pieces with root radii of 0.05 mm and 0.63 mm in as-built and stress-relieved states. Details at a higher magnification are shown in windows.

The fracture surface of the un-notched test piece tested in an as-built state (Figure 17a) is mainly dominated by the brittle mode of fracture with a small amount of smooth area besides the dimples, microvoids and tear ridges. The fracture surfaces of notched test pieces in the as-built state (Figure 17b,c) show higher dimples and sharp cleavage fractures that were produced due to the higher axial stress to deform the material. However, the different notch radii used does not significantly influence the fracture surface morphology for both the as-built (Figure 17b,c) and the stress-relieved state. The SEM fractographs of the stress-relieved test pieces are presented in Figure 18a–c.

The investigation of the un-notched test pieces tested in the stress-relieved state revealed an almost smooth fracture surface (Figure 18a). Several grain boundary cracks were observed, which indicates the presence of an intergranular fracture mechanism. As expected, the fracture surfaces revealed a dominant ductile mode of fracture with an increased quantity of dimples, some amount of cleavage facets and a finer fibrous microstructure than in the case of the as-built test pieces. The higher ductility caused the decrease of strength and the higher plastic deformation. Moreover, the investigation of the fracture surface of the notched test pieces tested in a stress-relieved state revealed a finer dimple fracture mechanism compared with the un-notched test pieces in the as-built state, indicating that the IN 625 superalloy is not prone to tensile brittleness induced by the presence of V-notches.

Future work will focus on the notch sensitivity of AM IN 625 built in other orientations, as well as on the assessment of high temperature influence on tensile notch sensitivity.

## 4. Conclusions

The additively manufactured IN 625 superalloy is not sensitive to tensile brittleness induced by the presence of V-notches. The material exhibits notch strength ratios higher than unity in both as-built and stress-relieved states, showing the lack of the notch sensitivity of the material in the presence of stress concentrators, such as V-notches.

The higher plasticity of stress-relieved materials result in lower levels of tensile strength than in the as-built state, associated with higher plastic deformations under tensile loads and elongations at fracture. Higher tensile strength recorded for the as-built material is caused by the microstructural characteristics consisting in highly textured columnar grains and very fine dendritic structures induced by the LPBF manufacturing process. Moreover, the deformation and elongation at the break are dependent on the notch radius—both elongation and deformation increase as the notch radius increases.

The higher strength of the V-notched test pieces as compared with the smooth test pieces is governed by the hardening effect in the cross section where the notch is located. Due to the higher triaxiality of stresses induced by notches, for both as-built and stress-relieved states, the proof of the strength of the notched test pieces is even higher than the tensile strength of the smooth test pieces in the same state.

The conclusions of ANOVA show that a very large part (91.5%) of the variation observed in the tensile strength data is due to the specimen’s condition. The influence of the stress concentrator factor is much smaller, but still significant (7.1%). If the influence of the specimen condition is removed by splitting the population according to it, the statistical significance of the stress concentrator factor becomes very large (*p*-value of the order of 10^−26^, respectively 10^−24^ on the two populations). Thus, the correlation between Kt and Rm is shown to be of medium strength for both specimen’s conditions (0.35, respectively 0.44).

The fractographic investigation revealed a mixed mode of ductile and brittle fracture of the tensile test pieces. The different notch root radii used does not noticeably influence the fracture surface morphology of the specimens tested in the same state, the as-built or stress-relieved state. However, for stress-relieved test pieces, there was encountered a more dominant ductile mode of fracture, with an increased quantity of dimples, some amount of cleavage facets and a finer fibrous microstructure than in the cases of the as-built test pieces.

These experimental results come to complete the overall picture regarding the notch sensitivity of the additive manufactured metallic alloys in general, and for the IN625 superalloy in particular. The strengthening effect of circumferential notches observed here may contribute to validate and calibrate the parameters in yielding or failure criteria for AM IN 625 material in service under tensile stresses. These findings can help the designers of complex additive manufacturing geometries to better predict the failure of their components and potentially identify suitable corrective actions.

Of course, it is not the intention of this study to be exhaustive and many other questions related to the influence of different heat treatments on the microstructure, testing environments (temperature, oxidative, corrosive) and more complex loading that can be encountered in operation, still exist and should be addressed.

## Figures and Tables

**Table 1:** Chemical composition of IN 625 metal powder (in wt. %).

Element	Al	C	Co	Cr	Fe	Mn	Mo	Nb	Si	Ti	Ni
Specification	<0.4	<0.1	<1.0	20–23	3–5	<0.5	8–10	3.15–4.15	<0.5	<0.4	Bal.
Actual composition	0.06	0.02	0.1	20.7	4.1	0.01	8.9	3.77	0.01	0.07	Bal.

**Table 2:** Stress concentration factor and notch strength ratio of as-built material.

Notch Root Radius, ρ, mm	0.05	0.06	0.16	0.15	0.32	0.33	0.65	0.65
*K_t_*	10.312	9.718	6.260	6.454	4.842	4.807	3.645	3.666
NSR	1.49	1.49	1.46	1.49	1.50	1.53	1.50	1.50

**Table 3:** Stress concentration factor and notch strength ratio of stress-relieved material.

Notch Root Radius, ρ, mm	0.05	0.05	0.17	0.17	0.32	0.32	0.68	0.68
*K_t_*	10.612	10.61	6.102	6.123	4.811	4.853	3.631	3.634
NSR	1.41	1.43	1.38	1.41	1.45	1.44	1.42	1.46

## Appendix A

**Table A1:** Tensile test results on as-built material.

No.	Notch Root Radius *ρ*, mm	Cylindrical Diameter d_1_, mm	Initial Diameter d_0_, mm	Diameter at Fracture d_0f_, mm	Tensile Strength Rm, MPa	Proof Strength R_p0.2_, MPa
1	0.05	7.448	5.280	5.080	1286	1164
2	0.06	7.520	5.240	4.958	1290	1170
3	0.16	7.510	5.297	5.000	1265	1134
4	0.15	7.515	5.284	5.028	1290	1170
5	0.32	7.480	5.118	4.885	1294	1153
6	0.33	7.500	5.109	4.963	1325	1187
7	0.65	7.490	5.217	4.790	1295	1116
8	0.65	7.520	5.210	4.909	1297	1136
9	∞	5.010	5.010	4.373	864	681

**Table A2:** Tensile test results on stress-relieved material.

No.	Notch Root Radius *ρ*, mm	Cylindrical Diameter d_1_, mm	Initial Diameter d_0_, mm	Diameter at Fracture d_0f,_ mm	Tensile Strength Rm, MPa	Proof Strength R_p0.2_, MPa
1	0.05	7.515	5.205	4.895	1165	1003
2	0.05	7.501	5.192	4.972	1175	1010
3	0.17	7.512	5.260	4.908	1139	996
4	0.17	7.525	5.268	4.984	1163	1004
5	0.32	7.500	5.169	4.857	1198	1005
6	0.32	7.520	5.174	4.862	1189	1007
7	0.68	7.514	5.153	4.797	1172	967
8	0.68	7.502	5.140	4.835	1206	983
9	∞	5.017	5.017	3.982	824	583

## Appendix B

**Table A3:** Input data for ANOVA: Two-Factor without replication and ANOVA: Single Factor (Rm).

α = 0.05					
Kt	Rm		Sum	Average	Variance
	As-Built	Stress-Relieved			
10.31235738	1286	1164.7	2450.7	1225.35	7356.845
9.717797887	1290	1175.01	2465.01	1232.505	6611.35
6.259515187	1265	1139.39	2404.39	1202.195	7888.936
6.454050483	1290	1163.23	2453.23	1226.615	8035.316
4.842199891	1294	1197.94	2491.94	1245.97	4613.762
4.806692671	1325	1188.8	2513.8	1256.9	9275.22
3.644588088	1295	1172.41	2467.41	1233.705	7514.154
3.666025564	1297	1205.94	2502.94	1251.47	4145.962
As-built			10.342	1292.75	270.7857
Stress-relieved			9407.42	1175.928	455.2126

**Table A4:** ANOVA: Two-Factor without Replication.

Source of Variation	Sum of Squares (SS)	Degrees of Freedom (df)	(Mean Squares) MS	F-Ratio (F)	Probability Value (*p*-Value)	F Critical	SS%
Rows	4230.429	7	604.347	4.967863	0.025356	3.787044	7.089474
Columns	54,589.99	1	54,589.99	448.7415	1.31 × 10^−7^	5.591448	91.48346
Error	851.5592	7	121.6513				1.427067
Total	59,671.97	15					

**Table A5:** ANOVA: Single Factor (Rm).

Source of Variation	Sum of Squares (SS)	Degrees of Freedom (df)	(Mean Squares) MS	F-Ratio (F)	Probability Value (*p*-Value)	F Critical	SS%
Between Groups	54,589.99	1	54,589.99	150.386	7.08 × 10^−9^	4.60011	91.48346
Within Groups	5081.988	14	362.9992				
Total	59,671.97	15					

**Table A6:** Input data for ANOVA: Single Factor (Kt).

Kt Gropus	Rm Sum (As-Built + Stress-Relieved)	Average	Variance
3–5	9976.09	1247.011	3734.388
5–7	4857.62	1214.405	5506.863
7–9	0	0	0
9–11	4915.71	1228.928	4673.13

**Table A7:** ANOVA: Single Factor (Kt).

Source of Variation	Sum of Squares (SS)	Degrees of Freedom (df)	(Mean Squares) MS	F-Ratio (F)	Probability Value (*p*-Value)	F Critical
Between Groups	2991.282	3	997.094	0.211097	0.886778	3.490295
Within Groups	56,680.69	12	4723.391			
Total	59,671.97	15				

**Table A8:** ANOVA: Single Factor As-built condition.

Groups	Sum	Average	Variance				
Kt	49.70323	6.212903	6.584174				
Rm As-built	10,342	1292.75	270.7857				
**Source of Variation**	**Sum of Squares** **(SS)**	**Degrees of Freedom (df)**	**(Mean Squares) MS**	**F-ratio (F)**	**Probability Value** **(** ***p*-value)**	**F Critical**	**SS%**
Between Groups	6,620,711	1	6,620,711	47,739.22	3.9 × 10^−26^	4.60011	99.97068
Within Groups	1941.589	14	138.6849				
Total	6,622,652	15					

**Table A9:** ANOVA: Single Factor Stress-relieved.

Groups	Sum	Average	Variance				
Kt	49.70323	6.212903	6.584174				
Rm Stress-relieved	9407.42	1,175,928	455.2126				
**Source of Variation**	**Sum of Squares** **(SS)**	**Degrees of Freedom (df)**	**(Mean Squares) MS**	**F-Ratio (F)**	**Probability Value** **(*p*-value)**	**F Critical**	**SS%**
Between Groups	5,472,929	1	5,472,929	23,702.76	5.23 × 10^−24^	4.60011	99.94097
Within Groups	3,232,578	14	230.8984				
Total	5,476,162	15					

**Table A10:** Kt—Rm correlation for the two populations (discriminated by the specimen condition).

	Kt	As-Built
Kt	1	
As-built	−0.34825	1
	**Kt**	**Stress-relieved**
Kt	1	
Stress-relieved	−0.44278	1
